# Artificial Intelligence in Minimally Invasive and Robotic Gastrointestinal Surgery: Major Applications and Recent Advances

**DOI:** 10.3390/jpm16020071

**Published:** 2026-01-31

**Authors:** Matteo Pescio, Francesco Marzola, Giovanni Distefano, Pietro Leoncini, Carlo Alberto Ammirati, Federica Barontini, Giulio Dagnino, Alberto Arezzo

**Affiliations:** 1 Department of Surgical Sciences, Università Degli Studi di Torino, Corso Dogliotti 14, 10126 Turin, Italy; matteo.pescio@unito.it (M.P.); francesco.marzola@unito.it (F.M.); giovanni.distefano@unito.it (G.D.); pietro.leoncini@polito.it (P.L.); carloalberto.ammirati@gmail.com (C.A.A.); federica.barontini@unito.it (F.B.); giulio.dagnino@unito.it (G.D.); 2 Department of Mechanical and Aerospace Engineering, Politecnico di Torino, Corso Duca degli Abruzzi 24, 10129 Turin, Italy; 3 Robotics and Mechatronics, University of Twente, Drienerlolaan 5, 7522 Enschede, The Netherlands

**Keywords:** surgical AI, robotic surgery, surgical innovation, surgical simulation, computer vision in surgery, surgical data science, surgical robot autonomy

## Abstract

Artificial intelligence (AI) is rapidly reshaping gastrointestinal (GI) surgery by enhancing decision-making, intraoperative performance, and postoperative management. The integration of AI-driven systems is enabling more precise, data-informed, and personalized surgical interventions. This review provides a state-of-the-art overview of AI applications in GI surgery, organized into four key domains: surgical simulation, surgical computer vision, surgical data science, and surgical robot autonomy. A comprehensive narrative review of the literature was conducted, identifying relevant studies of technological developments in this field. In the domain of surgical simulation, AI enables virtual surgical planning and patient-specific digital twins for training and preoperative strategy. Surgical computer vision leverages AI to improve intraoperative scene understanding, anatomical segmentation, and workflow recognition. Surgical data science translates multimodal surgical data into predictive analytics and real-time decision support, enhancing safety and efficiency. Finally, surgical robot autonomy explores the progressive integration of AI for intelligent assistance and autonomous functions to augment human performance in minimally invasive and robotic procedures. Surgical AI has demonstrated significant potential across different domains, fostering precision, reproducibility, and personalization in GI surgery. Nevertheless, challenges remain in data quality, model generalizability, ethical governance, and clinical validation. Continued interdisciplinary collaboration will be crucial to translating AI from promising prototypes to routine, safe, and equitable surgical practice.

## 1. Introduction

AI has begun to profoundly impact surgery, influencing everything from pre-operative planning and training to intraoperative guidance and postoperative outcomes [1,2,3,4]. Gastrointestinal (GI) surgery encompasses a high volume of procedures, for instance, colorectal cancer screening via colonoscopy and laparoscopic abdominal operations, which generate vast amounts of visual and clinical data [5,6,7,8,9]. This abundance of data makes GI surgery a fertile ground for AI applications [10,11,12,13]. Recent AI techniques are being leveraged to enhance surgeons’ capabilities. Supervised learning (SL) is adopted in computer vision (CV) to improve the detection of GI lesions [14], to optimize surgical decision-making [15], and for patient-specific surgical simulation [16]. Reinforcement learning (RL) and imitation learning (IL) are investigated for workflow automation [17] and augmented dexterity in robotic surgeries [18]. Recent studies indicate that AI can enhance colorectal cancer screening by augmenting endoscopists’ visual detection of precancerous polyps and improving the classification of lesions [19]. Likewise, machine learning (ML) models are now matching or exceeding traditional statistical models in predicting GI surgical outcomes, aiding in risk assessment and tailored patient management [20].

### Search Strategy and Study Selection

**Figure 1:**
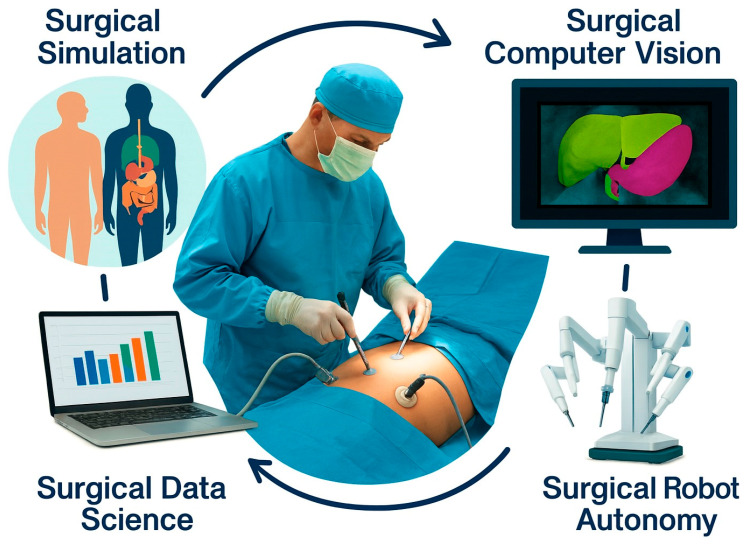
Overview of the major applications and recent advances of artificial intelligence in minimally invasive and robotic gastrointestinal surgery provided in this review. The chapters discuss the applications of AI for surgical simulation (Section 2), surgical computer vision (Section 3), surgical data science (Section 4), and surgical robot autonomy (Section 5).

Based on our expertise, we identified four major domains of AI application in surgery (Figure 1): Surgical Simulation, Surgical Computer Vision, Surgical Data Science, and Surgical Robot Autonomy. With this review, we highlight the state-of-the-art developments, current challenges, and potential research directions. In each section, we maintain discussions of core AI methodologies (e.g., digital twins, computer vision algorithms, workflow recognition, autonomy levels) but anchor them to GI surgery use cases, such as laparoscopic cholecystectomy, colorectal surgery, endoscopic interventions, and GI cancer surgery.

**Figure 2:**
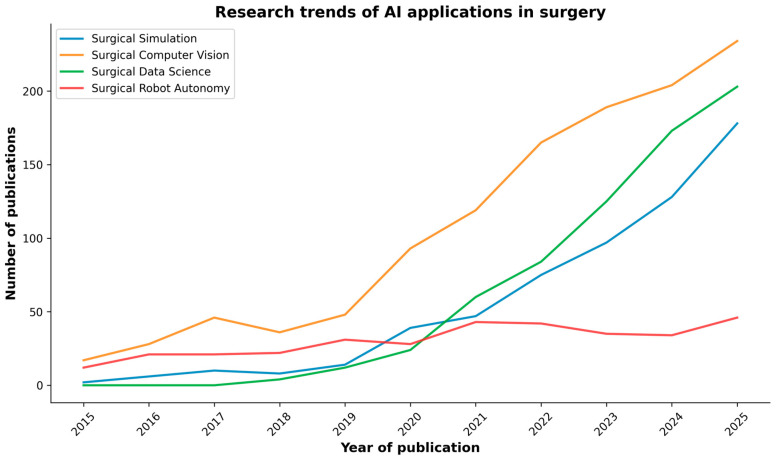
PubMed search results of publications on AI in surgery over the past decade, categorized by the major AI domains discussed in the review.

As research in AI applications in surgery is rapidly increasing (Figure 2), a review of key research studies was conducted across reputable academic databases, including PubMed, IEEE Xplore, and Google Scholar. The search was conducted using keywords and phrases such as “Artificial Intelligence”, “Simulation”, “Computer Vision”, “Data Science”, “Robot Autonomy”, and “Gastrointestinal Surgery”. These terms were used to identify relevant research articles, reviews, and conference papers written in English within the last decade (2015–2025). Occasionally, for very recent works accepted at major conferences, we also included preprint papers from open databases such as arXiv and TechRxiv. Two independent reviewers evaluated the titles and abstracts of the identified articles to assess their relevance. Subsequently, the entire articles were reviewed based on the inclusion criteria. Any discrepancies were resolved through discussions involving all the authors.

It is crucial to understand that this narrative review does not intend to provide a comprehensive taxonomy of research publications, and no formal PRISMA workflow, quality scoring, or meta-analysis was performed. On the contrary, this article presents a comprehensive perspective on AI in minimally invasive and robotic gastrointestinal surgery by integrating four major domains within a unified framework. While summarizing recent advancements, we identify recurring challenges (data, validation, and governance) and provide a synthesis (Table 1) to guide future translational research.

Through these examples and recent studies, we illustrate how surgical AI is transforming training, intraoperative guidance, data-driven decision support, and robotic assistance in the GI domain.

## 2. Surgical Simulation

The integration of AI into surgical simulation (Table 1) is shifting the field from isolated task trainers toward data-driven coaching, automated assessment, and patient-specific digital twins. In clinical education and perioperative planning, particularly in hepatobiliary and colorectal surgery, AI-enabled tools are being deployed to enhance preparation and decision-making [16]. A virtual-reality (VR) trainer paired with an AI assessor has demonstrated face, content, and construct validity for early psychomotor skills, indicating that scalability and objectivity can coexist from the outset [21]. Beyond these foundations, VR-augmented differentiable simulations allow surgeons to co-design operative plans while a physics-constrained optimizer maintains physiological plausibility, offering an early view of interactive, twin-based planning workflows [22]. Layered onto simulation, AI provides adaptive feedback and supports governance and reporting standards [16,23], while digital-twin-assisted surgery frameworks specify data flows, validation steps, and ethical considerations for both training and intraoperative navigation [24,25].

In simulated skills training, AI-supported instruction can be advantageous relative to traditional expert tutoring. An AI coach has matched or improved learning efficiency compared with expert instruction [26], and augmenting human teaching with AI analytics has yielded superior acquisition and retention on procedural tasks [27]. When AI-assisted video analysis is coupled to structured coaching for simulated laparoscopic cholecystectomy, novice performance and safety endpoints improve without loss of efficiency, with high acceptability among trainees [28]. Systematic reviews confirm that simulated curricula, especially those providing continuous motion feedback, enhance global ratings and shorten operative time, supporting adoption at scale [29,30]. Complementing these data, a randomized comparison of real-time, multifaceted AI with in-person instruction shows comparable or better skill acquisition with the AI tutor, pointing to more equitable access where faculty bandwidth is limited [31].

The Virtual Operative Assistant demonstrated that explainable-AI scores can align with expert ratings, accelerating deliberate practice while preserving interpretability, an essential prerequisite for clinical acceptance [32]. More recent work introduces larger simulated-laparoscopy datasets and deep architectures that discriminate skill levels with strong performance, reducing annotation burden and enabling objective program-level evaluation [33]. In parallel, deep learning models trained on simulator kinematics can estimate bimanual expertise in real time, yielding granular learning trajectories that drive adaptive coaching loops [34].

AI is likewise transforming patient-specific modeling for rehearsal and planning. In rectal cancer, an AI pipeline can generate detailed three-dimensional pelvic reconstructions directly from MRI, supporting preoperative simulation and spatial understanding [35]. In hepatic surgery, AI improves the extraction of portal and hepatic venous anatomy from CT to support virtual hepatectomy and remnant assessment [36], and end-to-end pipelines now produce comprehensive anatomical models that also predict resection complexity, informing multidisciplinary decision-making [37]. A randomized comparison of AI-accelerated segmentation for 3D-printed models versus digital simulation alone reports in preparation efficiency and selected intraoperative outcomes [38]. For complex hepatic procedures, mixed reality and AI are being integrated to enhance precision and safety, while exposing practical challenges in registration, workflow integration, and privacy that demand rigorous validation [25].

These developments converge toward dynamic, interactive simulations that link preoperative imaging with physics-based constraints [22,39], and toward digital twins for organ transplantation that simulate postoperative regeneration and functional recovery to personalize risk counseling and follow-up [40]. Finally, simulation environments underpin progress in surgical robotics: reinforcement learning benchmarks spanning spatial reasoning, deformable manipulation, dissection, and threading are lowering barriers to research on autonomy [41,42], while photorealistic digital twins and curated demonstrations enable behavior-cloning policies to master contact-rich subtasks such as needle handling and suturing, with systematic benchmarks across RGB and point-cloud visual inputs [43].

## 3. Surgical Computer Vision

Surgical computer vision (Table 1) has progressed from isolated prototypes to integrated systems that perceive the operative scene, document safety, assess skill, and begin to anticipate next actions in clinically meaningful ways. EndoNet established the feasibility of multi-task recognition (instruments and phases) from laparoscopic video and seeded today’s long-horizon models, while early phase-recognition pipelines on endoscopic video clarified how label design and temporal context affect real-world performance [44,45]. The CholecInstanceSeg dataset standardized instance-level tool segmentation for laparoscopic cholecystectomy and provided recommended splits and baselines to enable fair cross-study comparison [46]. In laparoscopic gastrectomy, convolutional neural networks (CNNs) reliably detected instruments and generated usage heatmaps [47]. Subsequent systems achieved real-time tool localization with clinically acceptable latency [48]; colorectal instrument-recognition models broadened coverage beyond cholecystectomy [49]; and a multi-hospital platform recognized instruments across five laparoscopic procedures and offered heatmaps, rapid review, and reporting that surgeons rated useful for quality improvement and education [50]. At the same time, deployment studies cautioned that single-site models may degrade when transferred across hospitals, devices, or surgical contexts, underscoring the need to plan for domain shift, diversify training data, and perform external validation [51].

Video platforms can localize critical events, such as the timing of cystic duct division, and extract concise clips to support objective reporting during laparoscopic cholecystectomy [52]. Pixel-level segmentation of the hepatocystic region combined with image-based classification of the Critical View of Safety (CVS) criteria shows that assessment can move beyond retrieval to evaluate whether the view is truly “critical” [53]. Other groups have trained CNNs to predict each CVS criterion directly from still images, demonstrating feasibility and highlighting inter-rater variability to address in prospective studies [54]. Near real-time intraoperative scoring systems can provide live feedback correlated with expert judgment, positioning AI as a natural adjunct to the pre-clipping safety check [55].

Anatomy-centric models now operate near real time and, for certain structures, can match or exceed the performance of most human raters; importantly, these studies candidly enumerate blind spots where anatomy is intermittently obscured [56]. By highlighting loose connective tissue, the dissectable layer, across gastrointestinal procedures, AI overlays can render “safe planes” more explicit [57]. In parallel, multi-center datasets and pipelines now detect and segment surgical gauze in liver surgery, mitigating a persistent source of error for both humans and algorithms [58].

Surgical workflow analysis has expanded across procedures. Step or phase recognition systems trained on full-length videos have been reported for transanal total mesorectal excision, laparoscopic hepatectomy, and totally extraperitoneal hernia repair, demonstrating robust parsing of complex operations in diverse fields [59,60,61,62]. In cholecystectomy, both classic CNNs and more recent long-video transformers capture extended temporal dependencies, improving recognition of transition-heavy segments where short windows struggle [62,63,64]. In gastric cancer surgery, phase-based metrics derived from recognition models correlate with expert skill scores; independent instrument-trajectory analyses further suggest that experts achieve shorter travel distances and more regular motion despite similar velocities [65,66].

A framework for simultaneous polyp and lumen detection during lower-GI endoscopy stabilized camera behavior and supported semi-autonomous navigation on a soft robotic colonoscope, supported by newly released real and phantom datasets [67]. Related work showed that CNNs can extract geometric features of the gastrointestinal tract from wireless-capsule imagery [68], and experimental studies demonstrated that narrowly scoped classifiers can provide reliable, explainable feedback in constrained settings such as laparoscopic Nissen fundoplication [69]. Finally, multimodal models, including large language models (LLMs), vision–language models (VLMs), and vision–language–action models (VLAs), are emerging as frameworks for surgical intelligence: modular agents can reason about workflow, decisions, and uncertainty from video and text [70]; LLMs can infer the next operative step from rich near-history context [71]; and, in VLAs, a dual-phase policy (perception–action) has performed autonomous tracking tasks, following lesions and adhering to cutting markers with interpretable intermediate reasoning and encouraging zero-shot generalization [72]. These higher-level systems will only be as reliable as their inputs; here, synthetic-to-real strategies are promising. For example, a reproducible pipeline for photorealistic robotic suturing produced instance-level labels at scale and improved instance segmentation when mixed with limited real data, an attractive approach for niche tasks with expensive annotation [73].

## 4. Surgical Data Science

Surgical data science (Table 1) is emerging as a disciplined, assistive layer that shapes performance, decisions, and team coordination throughout the perioperative pathway. In bariatric surgery, case-specific technical skill quantified from operative video correlated with excess weight loss at six to twelve months, linking intraoperative performance to patient outcomes and elevating technical skill from a proxy to a prognostic variable [74]. In colorectal surgery, machine learning (ML) models improved 30-day readmission prediction over conventional approaches and identified actionable contributors, including ileus, organ-space infection, and ostomy placement, thereby supporting targeted post-discharge pathways [75]. Real-time prediction of duodenal stump leakage during gastrectomy further illustrates how streaming perioperative signals can be converted into early warnings, enabling mitigation before biochemical or radiologic confirmation [76].

AI-based video analytics can also quantify how the evolving surgical field tracks expert standards, achieving high discrimination for skill classification and supporting objective, reproducible feedback loops for training and quality improvement [77]. Multi-institutional efforts emphasize reliability and fairness so that performance feedback remains consistent across sites and user groups, an essential prerequisite for broad deployment [78]. Beyond assessment, predictive tools have been developed to estimate case duration for common general and robot-assisted procedures, improving block allocation, on-time starts, and downstream staffing and bed management [79,80]. In parallel, pipelines for automated skill assessment have matured; for example, a three-stage video algorithm separates good from poor skill with high accuracy, laying the groundwork for finer-grained metrics as datasets expand in size and diversity [81]. Disease-specific perioperative models are also proliferating in gastrectomy, as early-complication predictors flag patients for targeted surveillance [82], while deep learning applied to intraoperative video has been leveraged to estimate colorectal anastomotic leakage risk in real time, potentially informing intraoperative and early postoperative decisions [83].

At the systems level, AI has been integrated with the Internet of Medical Things to collect, process, and analyze real-time vital signs during surgical and interventional care, creating pipelines that can deliver timely, interpretable alerts to clinicians [84]. Within anesthesia, a predictive ML system has been shown to warn of impending hypotension up to 20 min in advance, shifting management from reactive to anticipatory and aiming to reduce cumulative hypotension exposure [85]. Temporal models extend into the postoperative period as well: a recurrent neural network outperformed logistic regression for predicting colorectal postoperative complications, including wound and organ-space infections, supporting its potential use as a bedside risk-assessment tool with continuously updated estimates [86].

Random-forest models predict anastomotic leakage after anterior resection for rectal cancer, highlighting predictors that align with surgical judgment [87]. Phase-recognition models, long used for workflow analysis, now serve as substrates for skill assessment in laparoscopic cholecystectomy, aligning with expert ratings and stratifying proficiency levels [88]. In hepatobiliary surgery, classifiers for liver-resection complications achieve high discrimination while surfacing practical predictors, such as operative duration, body mass index, and incision length, that naturally integrate with prehabilitation and intraoperative planning [89]. Similarly, decision-tree and gradient-boosting models have been constructed to predict postoperative ileus after laparoscopic colorectal carcinoma surgery, pointing toward parsimonious, modifiable risk bundles [90]. Finally, an AI-based perioperative safety-verification system improved the execution and standardization of surgical checklist steps in general surgery, underscoring how thoughtfully embedded decision support can enhance team reliability without disrupting clinical flow [91].

## 5. Surgical Robot Autonomy

Recent research on surgical robot autonomy (Table 1) aims to increase the levels of autonomy and assistance achievable in the operating room (OR) [92]. Despite steady progress, most commercial systems are still classified as Level 0 (i.e., no autonomy), underscoring the translational gap between research prototypes and deployed platforms [93,94]. Early landmark demonstrations in soft-tissue anastomosis showed that supervised autonomy can match or surpass expert performance on precision and consistency, reframing the clinical question from “whether” to “where” autonomy is safely useful [95]. Subsequent in vivo studies, incorporating vision-guided self-corrections, further demonstrated that delicate suturing can be performed reliably if perception, planning, and safety monitors are tightly integrated [96]. Endoluminal platforms have, in parallel, articulated staged “intelligence layers” from teleoperation to semi-autonomy, showing that even non-experts benefit when navigation or viewpoint management is delegated to intelligent controllers [17].

Visualization and exposure are natural entry points for autonomy because they relieve routine burdens without displacing surgical judgment. Instrument-aware visual servoing can center tools and maintain task-appropriate viewpoints while allowing smooth handover to the surgeon when needed, improving coordination without eroding trust [97]. Extending this beyond rigid scopes, a tendon-driven flexible endoscope coupled with optimal control tracked instruments while minimizing both internal and external workspace occupation, addressing safety and ergonomics central to minimally invasive procedures [98]. Learning-based camera assistance has also matured: a cognitive controller that improves with experience, reduces operative time, and increases the proportion of “good” views across sessions [99]. On the da Vinci Research Kit (dVRK) [100], a voice-enabled autonomous camera achieved workload and user-preference profiles comparable to a human operator despite modest differences in completion time [101]; likewise, a purely vision-based RL agent mapped endoscopic images directly to camera actions to maintain centering without additional sensors [102]. Most recently, robotic camera holders for laparoscopy have shown rapid response while respecting the remote center of motion (RCM) with favorable usability ratings, suggesting practical readiness for hands-free camera assistance in standard tasks [103].

Beyond visualization, autonomy is being explored for intraoperative support. Blood suction, a frequent cause of pauses and errors, is an attractive target: investigators transferred an RL policy trained in a particle-based fluid simulator to physical setups, reliably evacuating clinically meaningful volumes [104]. Building on this, multimodal large language models have acted as high-level planners that sequence suction primitives over long horizons, generalizing across tools and bleeding conditions and hinting at how language can couple perception, intent, and action in the OR [105]. For tissue exposure, demonstration-guided RL produced reliable soft-tissue retraction with safeguards that respect tissue mechanics [106], while pixel-space domain adaptation enabled the first successful visual sim-to-real transfer for deformable-tissue retraction without retraining, an important milestone for data efficiency and robustness [107]. Complementary work trained RL agents to learn optimal tensioning policies and action sequences for soft tissue cutting, addressing a well-known source of variability during dissection [108].

Suturing remains the principal proving ground because it couples perception, dexterity, planning, and error recovery into a single loop. Augmented-dexterity pipelines have achieved fully autonomous throws with 6-DoF needle pose estimation, thread coordination, hand-offs, and recovery primitives on the dVRK [109]. Iterative upgrades, extended Kalman filtering for needle tracking, 3D suture-line alignment, and improved thread management have yielded materially more throws in less time and higher wound-closure quality, especially when limited human interventions are permitted as guardrails [110]. Other efforts integrate finite-element modeling of tissue forces with deep networks for wound-edge detection and then execute sutures end-to-end on industrial arms fitted with surgical wrists, illustrating how physics-aware reasoning can raise reliability and safety margins [111]. At the skill-composition level, RL has automated the surgeon–assistant needle hand-off with sparse rewards while preserving surgeon-like trajectories and robustness to variable starting conditions, a micro-policy that plugs into larger suturing pipelines [112]. More recently, a diffusion-model-based framework combined with dynamic time warping-based locally weighted regression achieved high success rates in both simulated and real settings, advancing long-horizon autonomy under variability [113]. To standardize evaluation and prevent metric drift, a benchmark with substantial demonstrations and goal-conditioned assessment has been introduced, showing sizable gains in insertion-point accuracy over task-only baselines across newer multimodal AI models [114].

IL foundations have progressed in parallel to support multi-task behaviors. Transformer-based architectures cast low-level surgical manipulation as sequence modeling, enabling the learning of dexterous skills and their composition across tasks, such as needle picking, knot tying, and tissue retraction on the dVRK [115,116]. Extending this paradigm, a hierarchical, language-conditioned framework was introduced: a high-level policy selects the subtask while a low-level controller executes precise motions, enabling fully automated clipping-and-cutting in ex vivo cholecystectomy with high success on previously unseen specimens [117].

**Table 1:** For each section of the review, technical and clinical applications of surgical AI models and methods are reported.

Technical Application	Clinical Application	Data Modality	AI Model/Method	Outcome	References
**Surgical Simulation**					
Skill assessment	GI	VR, kinematics	Supervised scoring	Intraoperative expert tutoring	[21]
Planning	Colorectal	VR, medical imaging, digital twins	Anatomy segmentation	Improved efficiency, planning fidelity	[22,35,36,37,38,39,40]
Coaching, training	Laparoscopy, cholecystectomy	VR, telemetry	Analytics	Scalable coaching workflows	[23,24,25,26,27,28,29,30,31]
Autonomous task execution	GI	Image RGBD, kinematics	RL, IL	Benchmark for AI agents training	[41,42,43]
**Surgical Computer Vision**					
Scene understanding	Laparoscopy	Video RGB	CNN detection	Improved spatial understanding and contextualization	[44,45]
Tool segmentation	Laparoscopy, cholecystectomy	Video RGB	CNN segmentation	Improved spatial understanding and contextualization	[46,73]
Tool and object detection	Laparoscopy	Video RGB	CNN detection	Improved spatial understanding and contextualization	[47,48,49,50,58]
Unsafe event detection	Laparoscopy, cholecystectomy	Video RGB	CNN detection	Timing of critical steps	[51,52]
Safety verification	Critical view of Safety scoring	Video RGB	CNN data aggregation	Near real-time safety check	[53,54,55]
Anatomy segmentation	GI dissection	Video RGB	CNN segmentation	Enhanced intraoperative guidance	[56,57]
Anatomy segmentation	Colonoscopy	Video RGB	CNN detection	Enhanced intraoperative guidance	[67,68,69]
Workflow/Phase recognition	GI	Video RGB	Transformer temporal detection	Enhanced intraoperative guidance	[59,60,61,62,63,64,65,66]
Multi modal frameworks	GI	Video RGB, text, kinematics	LLM, VLM, VLA	Robust perception and planning	[70,71,72]
**Surgical Data Science**					
Outcome prediction	GI, bariatric, colorectal	Video RGB	RNN regression	Procedure outcome prediction	[74,86,87]
Outcome prediction	Colorectal	Perioperative data	Classification	Mid/Long term readmission prediction	[75]
Unsafe event prediction	GI, Gastrectomy	Perioperative data	Classification	Procedural events prediction	[76,82,83,84,85,89,90]
Skill classification and assessment	GI	Video, kinematics	CNN classification	Automated skill assessment	[77,78,81]
Case duration prediction	GI	Perioperative data	Regression	Automated case duration prediction	[79,80]
Safety verification system	GI	Video RGB, sensors	Regression	Perioperative unsafe events detection	[91]
**Surgical Robot Autonomy**					
Camera control	Laparoscopy, Endoscopy	Video, kinematics	RL, visual servoing, voice control	Autonomous tool-centering camera	[97,98,99,100,101,102,103]
Blood suction	Laparoscopy	Image RGBD, forces	RL, sim to real	Autonomous blood suction	[104,105]
Tissue retraction	GI	Video RGBD, forces	RL, sim to real	Autonomous organ exposure and tissue tensioning	[106,107,108,116]
Suturing	GI	Video RGB, kinematics	CNN segmentation, planning and control	Autonomous anastomosis	[95,96,109,110,111,112]
Surgical tasks	GI	Video RGB, kinematics	Diffusion models	Autonomous surgical subtasks	[113,114]
Surgical tasks	GI, ex-vivo cholecystectomy	Text, video RGB, kinematics	VLA, Transformers, Diffusion models	Hierarchical, Language-conditioned policy	[115,116]

## 6. Discussion

Artificial intelligence is revolutionizing surgery, impacting various aspects of surgical training, planning, execution, and evaluation. Over the past decade, particularly in the last five years (Figure 2), the field has transitioned from proof-of-concept studies to practical tools on the verge of routine use [1].

To translate these advances from research prototypes to routine practice in GI surgery, three cross-cutting challenges are particularly relevant: (i) multidisciplinary implementation and technical integration of heterogeneous data sources, (ii) robust validation and benchmarking that link technical metrics to patient- and system-level outcomes, and (iii) ethical and legal frameworks that keep pace with increasing levels of intelligence and autonomy.

### 6.1. Multidisciplinary Implementation and Integration Across Data Sources

Successful deployment of AI in GI surgery depends on close collaboration between surgeons, endoscopists, computer scientists, data engineers, ethicists, and hospital IT teams. Each of the four domains requires integration of multiple data streams and, increasingly, multi-omics and longitudinal follow-up data.

In surgical simulation, AI-enabled VR trainers, patient-specific 3D models, and early digital-twin frameworks will only reach their potential if they are embedded in formal curricula and institutional workflows. This implies standardized interfaces to hospital PACS and planning systems, automated ingestion of CT/MRI and endoscopic video, and alignment with competency-based training programs in surgery. Multidisciplinary teams must also define how simulation outputs feed back into decisions about credentialing and operative planning.

In surgical computer vision, clinically useful systems must be integrated with the existing OR stack. Real-time instrument recognition, anatomy overlays, and Critical View of Safety assessment need low-latency access to video and a robust path to display feedback without disrupting existing workflows. Co-development with OR nurses, biomedical engineers, and human-factor specialists is essential to ensure that feedback is presented in an intuitive and non-taxing way.

In surgical data science, predictive models require federated access to heterogeneous data sources. Implementing such models demands robust data pipelines, data-quality checks, and governance structures within hospitals and networks. Clinicians, data scientists, and IT departments must jointly determine how risk estimates are surfaced and how they are combined with existing risk scores to support decisions without overwhelming clinicians.

In surgical robot autonomy, shared control and autonomous systems must be interoperable with commercial robotic platforms and meet stringent safety and usability constraints. Implementation requires collaboration among OR personnel and regulatory experts to design interfaces that make autonomy transparent and overridable, and to ensure that autonomous behaviors can be tailored to different GI procedures.

### 6.2. Need for Robust Validation and Benchmarking

While many of the systems described in this review report impressive technical performance, current evidence is still dominated by single-center, retrospective, or simulation-based studies. To ensure reliable benefits in GI surgery, each domain requires systematic validation and standardized benchmarking [118].

For surgical simulation, most evidence relates to construct validity and short-term training outcomes. The next step is to design prospective, multi-center trials that test whether AI-enhanced simulation improves not only simulator metrics but also real-world performance. Benchmarking efforts should define common tasks, outcome measures, and follow-up intervals so that different simulators and AI coaching systems can be compared on a common scale.

In surgical computer vision, there is a growing ecosystem of datasets and benchmarks for tools, phases, anatomy, and safety cues. Robust translation into routine surgeries requires external validation across institutions, devices, and geographical regions, with explicit reporting of performance under domain shift. Common benchmark tasks should be associated with transparent releases of data splits, evaluation scripts, and clinically meaningful metrics to support fair comparison and cumulative progress.

For surgical data science, predictive models often achieve higher AUROC or calibration than traditional scores, but many remain at the proof-of-concept stage. Future studies should prioritize prospective validation, ideally with impact analyses that quantify how using these models changes management and outcomes. Benchmarking here should include not only discrimination and calibration but also decision-curve analysis, subgroup performance (e.g., by age, comorbidity, center), and robustness to missing data.

In surgical robot autonomy, many demonstrations are performed in benchtop or preclinical settings, using research platforms and synthetic tasks. To progress toward clinical deployment in GI surgery, the field needs standardized evaluation protocols, including task definitions, environmental conditions, and success criteria that link technical performance to surrogate clinical endpoints. Multi-center preclinical trials and, ultimately, carefully controlled first-in-human studies will be required to establish safety and efficacy, with pre-defined stopping rules and oversight by independent committees.

Beyond validation, the choice of model itself involves practical trade-offs that impact generalizability and deployability. In surgical computer vision, CNN-based detectors and segmenters excel when targets are primarily spatial (e.g., tools, anatomy, safety landmarks) and low latency is crucial. They often achieve robust real-time performance with moderate dataset sizes. On the other hand, Transformer-based long-video workflows are particularly advantageous for phase and workflow recognition, as well as higher-level understanding. This is because attention mechanisms can leverage long-range temporal context across minutes. However, they are typically more data and compute-intensive, and real-time deployment may necessitate pretraining, efficient attention, or model compression. In surgical data science, classical machine learning (e.g., regularized regression, random forests, gradient boosting) is often competitive for structured perioperative variables. This is because they offer simpler calibration and interpretability. In contrast, deep learning (e.g., RNNs/temporal transformers and multimodal models) becomes more valuable when raw time-series, waveforms, kinematics, or video are available, and manual feature engineering becomes a bottleneck. Therefore, a pragmatic direction for gastrointestinal surgery is to develop hybrid pipelines. These pipelines combine deep models to extract representations from unstructured signals with simpler, well-calibrated predictors for risk estimation. This approach aims to balance performance, transparency, and data requirements.

### 6.3. Ethical and Legal Considerations in GI Surgical AI

As AI systems in GI surgery progress from decision support to higher levels of autonomy, ethical and legal questions become central to their design and deployment. These considerations cut across simulation, computer vision, data science, and robot autonomy and must be addressed explicitly if AI is to be trusted as a clinical partner rather than perceived as a black box.

In surgical simulation, large datasets of surgical gestures, errors, and video recordings from surgeons raise questions about data ownership, consent, and the use of performance metrics. Institutions must define who can access simulation-derived performance data, for what purposes (education, credentialing, research), and how long such data may be retained. Surgeons should be informed when their data are used to train new AI models or to benchmark others. If simulation-based metrics are eventually used in formal certification or privileging, transparent criteria and appeals processes will be needed to avoid unfair or opaque decisions.

In surgical computer vision, the routine capture and analysis of high-resolution operative and endoscopic video for tool tracking, anatomy segmentation, or safety checks raises issues of privacy, annotation of identifiable content, and secondary use of video. De-identification of GI surgical video is non-trivial, as visual clues can reveal institutions, devices, and even patient characteristics. Governance frameworks must specify consent procedures for using operative video in research and commercial development, restrictions on cross-border data transfer, and safeguards against unintended uses. Moreover, biases in training datasets and underrepresentation of certain patient groups, disease stages, or centers may lead to systematically worse performance in those populations, which is ethically unacceptable for safety-critical tasks.

In surgical data science, models that assign risk scores for leak, readmission, or complications may influence decisions about postoperative monitoring, ICU admission, or even patient selection. This raises concerns about algorithmic fairness, explainability, and accountability. Surgeons and patients should understand, at least at a high level, what variables drive risk estimates and how uncertainties are handled. If AI-derived scores disproportionately classify specific groups (e.g., older or comorbid patients) as “high risk” based on biased training data, there is a risk of reinforcing inequities in access to complex GI procedures. Legal questions also arise: when recommendations from AI systems are followed, and adverse events occur, responsibility will be shared between clinicians, institutions, and manufacturers [119]. Clear guidance from regulators and professional societies will be required on how to document, audit, and govern the use of AI-powered decision support in perioperative care.

In surgical robot autonomy, ethical and legal stakes are particularly high. Shared-autonomy systems for camera control or suction are already influencing intraoperative actions, and more advanced systems for suturing or tissue manipulation may, in the future, execute parts of GI procedures with limited human intervention. Core questions include: who is accountable for an adverse outcome when autonomous actions contribute to a complication? What level of human oversight (e.g., always-in-the-loop vs. on-the-loop) is acceptable for different tasks? How should informed consent documents describe the role of autonomous functions in surgical procedures? Regulatory frameworks will likely differentiate between AI used as decision-support software, AI embedded in robotic platforms, and systems granting task-level autonomy, each with specific requirements for preclinical evidence, post-market surveillance, and incident reporting. From an ethical perspective, maintaining surgeon situational awareness and preserving the ability to override autonomous actions at any time will be important to maintain trust and uphold the surgeon’s primary responsibility toward the patient.

## 7. Conclusions

This review delves into the significant applications and recent advancements of artificial intelligence (AI) in gastrointestinal (GI) surgery. It covers various aspects, including surgical simulation, surgical computer vision, and surgical data science.

Each section explores the principal AI methodologies, such as digital twins, computer vision algorithms, workflow recognition, and autonomy levels. However, these methodologies are anchored to specific GI surgery use cases, including laparoscopic cholecystectomy, colorectal surgery, endoscopic interventions, and GI cancer surgery. Diagnostic AI applications, such as polyp detection and classification, have been introduced briefly but not extensively explored due to the existing extensive literature on the subject. Also, while AI models for predicting early postoperative outcomes and complications are discussed, AI applications for long-term postoperative follow-up, recurrence detection, or monitoring of recurrent symptoms are beyond the scope of this narrative review.

In GI surgery, AI holds promise for supporting organ-preserving pathways. It can refine patient selection for local excision and watch-and-wait strategies, optimize risk stratification, and standardize functional and oncologic outcome assessment. Additionally, computer vision and autonomous robotic tools have the potential to enhance the quality and reproducibility of surgical tasks, whether laparoscopic, robotic, or transanal. This improvement can be achieved by enhancing anatomical identification, dissection planes, and intraoperative feedback.

To realize this potential, several factors must be considered. Large, high-quality multicenter datasets are essential, along with shared benchmarks and metrics. Robust validation on clinically meaningful endpoints is crucial, and careful integration into surgical workflows, training programs, and regulatory frameworks is necessary. If developed and implemented responsibly, AI-enabled technologies can become a key enabler of safer, more standardized, and more personalized minimally invasive and robotic surgery. This has a particularly significant impact on rectal cancer care and organ-preserving strategies.

In the coming years, we anticipate the emergence of AI-driven surgical systems that continuously learn and improve, ultimately leading to safer surgeries, better patient outcomes, and more efficient utilization of healthcare resources in gastrointestinal surgery. The key applications and advancements will serve as the driving force behind this transformative journey, propelling us closer to the era of truly intelligent surgery that benefits both patients and surgeons. Despite these promising developments, significant obstacles remain in the widespread clinical adoption of AI, including high costs, technical complexity, limited generalization capabilities on unseen data, and the need for seamless integration into existing clinical workflows.

These technologies hold the potential to significantly enhance the accuracy, safety, and efficiency of gastrointestinal surgeries, ultimately leading to improved patient outcomes. To overcome the current challenges and fully realize the potential of surgical AI, continued collaboration between engineers, clinicians, and researchers is essential.

## Data Availability

All data are presented in this review article.

## Abbreviations

The following abbreviations are used in this manuscript:

AI	Artificial Intelligence
ML	Machine Learning
DL	Deep Learning
GI	Gastrointestinal
MR	Mixed Reality
VR	Virtual Reality
CV	Computer Vision
SL	Supervised Learning
RL	Reinforcement Learning
IL	Imitation Learning
RNN	Recurrent Neural Network
CNN	Convolutional Neural Network
LLM	Large Language Model
VLM	Vision Language Model
VLA	Vision Language Action Model
